# Superhydrophobic SLA 3D printed materials modified with nanoparticles biomimicking the hierarchical structure of a rice leaf

**DOI:** 10.1080/14686996.2022.2063035

**Published:** 2022-05-06

**Authors:** Belén Barraza, Felipe Olate-Moya, Gino Montecinos, Jaime H. Ortega, Andreas Rosenkranz, Aldo Tamburrino, Humberto Palza

**Affiliations:** a Matemáticas, Universidad de ChileDepartamento de Ingeniería Química, Biotecnología y Materiales, Facultad de Ciencias Físicas y, Santiago, Chile; b Núcleo Milenio en Metamateriales Mecánicos Suaves e Inteligentes (Millennium Nucleus on Smart Soft Mechanical Metamaterials); c Advanced Mining Technology Center, Universidad de Chile, Santiago, Chile; d Departamento de Ingeniería Matemática, Universidad de la Frontera, Temuco, Chile; e Departamento de Ingeniería Matemática, Facultad de Ciencias Físicas y Matemáticas, Universidad de Chile, Santiago, Chile; f Centro de Modelamiento Matemático, IRL 2807 CNRS-UChile, Facultad de Ciencias Físicas y Matemáticas, Universidad de Chile, Santiago, Chile; g Departamento de Ingeniería Civil, Facultad de Ciencias Físicas y Matemáticas, Universidad de Chile, Santiago, Chile

**Keywords:** 3D printing, biomimetic, hierarchical structure, rice leaf, superhydrophobic

## Abstract

The rice leaf, combining the surface properties of lotus leaves and shark skin, presents outstanding superhydrophobic properties motivating its biomimesis. We created a novel biomimetic rice-leaf superhydrophobic surface by a three-level hierarchical structure, using for a first time stereolithographic (SLA) 3D printed channels (100µm width) with an intrinsic roughness from the printing filaments (10µm), and coated with TiO_2_ nanoparticles (22 and 100nm). This structure presents a maximum advancing contact angle of 165° characterized by lower both anisotropy and hysteresis contact angles than other 3D printed surfaces, due to the presence of air pockets at the surface/water interface (Cassie-Baxter state). Dynamic water-drop tests show that the biomimetic surface presents self-cleaning, which is reduced under UV-A irradiation. The biomimetic surface further renders an increased floatability to 3D printed objects meaning a drag-reduction due to reduced water/solid contact area. Numerical simulations of a channel with a biomimetic wall confirm that the presence of air is essential to understand our results since it increases the average velocity and decreases the friction factor due to the presence of a wall-slip velocity. Our findings show that SLA 3D printing is an appropriate approach to develop biomimetic superhydrophobic surfaces for future applications in anti-fouling and drag-reduction devices.

## Graphical Abstract

**Figure:**
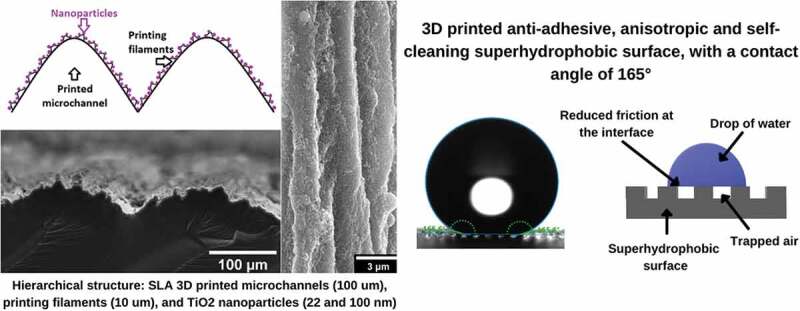

## 1. Introduction

Biomimesis aims to solve human problems by mimicking the solutions that nature has already found through the collaboration of different scientific fields, such as chemistry, biology, physics, nanotechnology, tribology, material sciences, and engineering. An example of this is the biomimicry of the topography of natural surfaces, which aims to reproduce the unique arrangement of hierarchical micro and nano-structures found in the surfaces of the lotus leaf [1], the rice leaf [2], and insect wings [3–5], among others. It has applications in the development of new materials with topographies that allow anti-biofouling, drag reduction, detection of analytes, and improved catalysis. Recent investigations report the use of nanoarchitectonics for the development of new biomimetic surfaces, which construct micro and nano-metric topographies through the manipulation of atoms and molecules [6].

One of the challenges solved by nature is the control of the interaction between water and a surface through superhydrophobic structures [7], allowing for a drag reduction in fluids [8]. Viscous drag and pressure resistance are the two main mechanisms by which objects resist the movement of fluids, or vice versa, playing a crucial role in the development of hydrodynamic structures. From the different contributions to the total drag force, viscous drag is the most relevant requiring the largest amount of energy to overcome [9]. Nature faces this problem using surfaces that either repel water (for laminar flows) or stabilize the flow behavior or vortices (for turbulent flows). For instance, the shark skin experiences drag reduction for turbulent flows due to the presence of denticles with riblets on their surfaces [10]. The specific surface morphology and superhydrophobicity found in nature can add other functionalities such as self-cleaning, which is found for the lotus leaf [1], the rice leaf, and butterfly wings [3]. These naturally occurring surfaces repel droplets of water without opposing any resistance to their movement due to the presence of air pockets at the interface, thus motivating a biomimetic design of materials to solve relevant industrial problems such as pollution, corrosion, icing, and biofilm formation [7,11–14].

**Figure 1.**
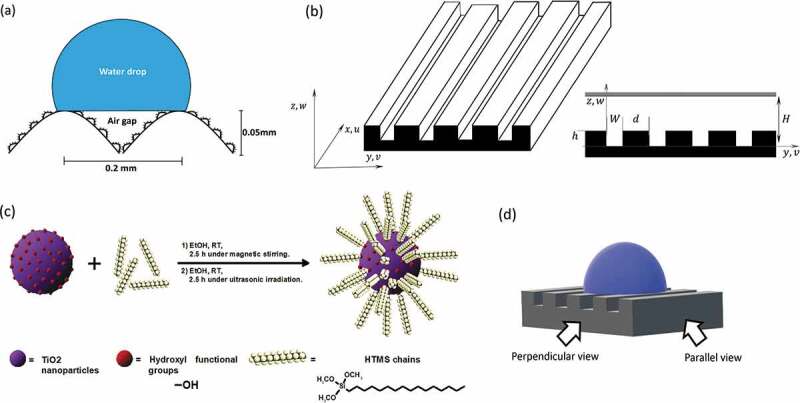
(A) Scheme of the hierarchical structure of the superhydrophobic rice leaf surface composed of micrometric riblets and hydrophobic papillae with nanometric features on top of them. This structure forms air pockets between the surface and the water droplet. (b) Geometry and coordinate axis of the biomimetic surface studied and simulated. Here, the flow moves through the x axis with a velocity u, while h represents the height of the microchannel, H stands for the gap between microchannels, W is their width (d), and h is the distance between the bottom and the top of the channel. (c) Silanization process of the TiO_2_ nanoparticles, where the HTMS chains attach via the hydroxyl functional groups and provide a hydrophobic behavior. (d) Perpendicular and parallel views of the surface for contact angle measurements orientation.

Superhydrophobicity is a type of wettability tending to repel a drop of water with contact angles above 150°, a contact angle hysteresis below 10° (difference between the advancing and receding angle), a sliding angle of less than 5°, and high stability in the Cassie-Baxter state (i.e. presence of air trapped between the surface and the water droplet) [15,16]. In nature, superhydrophobic surfaces have a hierarchical structure at the micro-and nanometric scale [17]. Regarding different natural materials featuring this behavior, the rice leaf is highlighted because it combines the properties of the lotus leaf and the shark skin [8], thus having a three-level hierarchical structure as depicted in Figure 1a. This structure is based on a micrometric arrangement of channels or riblets (around 50µm in height with a peak separation separated of 200µm) with rough papillae on top of them (around 2µm) covered by wax (changed surface chemistry) [18]. This hierarchical structure provides a superhydrophobic behavior with a contact angle of 164° and a sliding angle anisotropy of approximately 6° [2,8].

Hierarchical superhydrophobic surfaces can be manufactured using either physical modification (for instance by plasma and molding processes) [19,20], or hydrophobic coatings (for instance by spin-coating, aerosol, and electrochemical deposition) [21,22]. Apart from those processes, 3D printing has been gaining interest in the last years since it allows for the creation of objects with many possibilities in terms of precision, sizes, shapes, mechanical and surface properties, including from delicate prototypes to robust engineering products. By adding layers of a polymeric material, 3D printing can create micropatterns needed in the hierarchical structure of a superhydrophobic surface through techniques such as fused deposition modeling (FDM) [23–25], inkjet printing [26], one-step 3D printing [27], immersed surface accumulation-based 3D (ISA-3D) printing [28], and mold imprinting [2,3,8], among others. However, irrespective of the method used, the printed structure cannot reach a superhydrophobic wettability by itself as polymeric resins are mostly hydrophilic [29]. Moreover, they do not have a hierarchical structure, which directly asks for a surface modification such as the application of a nanostructured, hydrophobic coating. Titanium dioxide (TiO_2_) based coatings are studied since they can provide a superhydrophobic character to different materials such as cellulose sponges [30], polydimethylsiloxane (PDMS) [31], and flat glass pieces [32]. They can be applied by dip-coating, spraying, and spin-coating, among others [33]. Additionally, coatings consisting of TiO_2_ nanoparticles have been combined with other substances due to their photocatalytic, bactericidal, and self-cleaning properties [32,34–37]. Rice leaf biomimetic surfaces have also been developed using both nanostructured silica coatings and 3D printing including fused deposition modeling (FDM) and mold imprinting [8,23], although other coatings can also be used for this purpose (for example polydivinylbenzene powder) [2].

Regarding the printing process, stereolithographic (SLA) 3D printing has advantages over other 3D printing techniques regarding its deposition speed (10^5^ mm^3^h^−1^) and high precision (25µm) [38], besides being the oldest and, therefore, the most mature 3D printing techniques [39]. However, despite all these advantages, it has been barely used to manufacture biomimetic surfaces. Few examples can be found dealing with lotus leaves with a contact angle of 139° without any additional coating and shark skin with a drag reduction of 10% in pipe experiments [40], as well as a cactus surface coated with TiO_2_ nanoparticles having a contact angle below 160° [41]. A biomimetic surface based on a rice leaf is yet to be explored by SLA. Moreover, the use of the microstructure provided by the SLA 3D printing filaments for the construction of a biomimetic hierarchical structure has not been reported yet. Consequently, this study aims at mimicking the three-level hierarchical structure of the rice leaf using SLA 3D printed microchannels modified by the incorporation of TiO_2_ nanoparticles functionalized by hexadeciltrimethylsiloxane (HTMS). Our results show that besides the morphology of the designed 3D printed structure generated by SLA (of around 100µm), the imperfections from the presence of SLA 3D printed filaments (of around 10µm) render a hierarchical structure providing a further roughness to the printed surface, to which the nanoparticles (22 and 100nm) were added. The combination of SLA 3D printing and TiO_2_ coatings can be used in many applications, as it allows for the printing of infinite shapes with a biomimetic surface. Numerical simulations were further carried out to confirm the underlying mechanism of the dynamic superhydrophobic properties arising from air pockets that the biomimetic material provides.

## 2. Experimental methods

### 2.1. Mathematical model and numerical schemes

Superhydrophobic surfaces found in nature have special water-repellent properties when a Cassie-Baxter state exists (i.e. air gaps trapped between a drop of water and the surface) [42]. The air gaps endure when the surface is exposed to laminar flows of water, as for turbulent flows the pressure differences at the solid-water interface will probably eliminate the air gaps and penetrate the micrometric topography. Nevertheless, there is a wide consensus that riblets also allow drag reduction in turbulent flows [9,43–48], as can be observed in the shark skin [8]. These investigations also report that a superhydrophobic coating over a riblet geometry can increase drag reduction up to 10% at turbulent Reynolds numbers [9]. The drag reduction mechanism for turbulent flows over surfaces with riblets is different compared to laminar flows, as the micrometric structure stabilizes vortex formation without the existence of air gaps [8].

Numerical simulations were carried out to visualize the effect of the air pockets, considering a laminar water flow on the superhydrophobic biomimetic structure. For this purpose, the software FreeFem++ (v. 4.6) was used to solve the Navier–Stokes equations within a channel domain. FreeFem++ is a free and open-source software designed to solve partial differential equations (PDE) using Finite Element Method schemes (FEM) [49]. This method is widely used in engineering and science to numerically model fluid dynamics and multiphysics problems [50,51]. The material geometry and coordinate axis for the definition of the equations used in the numerical simulations are shown in Figure 1b.

The following aspects were considered for the numerical simulations: the fluid (water) will flow inside a closed rectangular channel, where only the bottom-wall surface has the biomimetic microchannel structure (riblet). The top wall will be hydrophilic, which translates to a no-slip boundary condition. The fluid will be water at ambient conditions (20°C 1atm), and the flow regimen will be laminar. The fluid flow direction will be parallel to the microchannels. The lateral walls of these rectangular channels presented periodic boundary conditions to replicate the periodic arrangement of the experimental biomimetic surface, so lateral boundary conditions are non-existent since they are not relevant to understanding the phenomena at the bottom wall. The air trapped between the microchannels is considered incompressible. The flow will be produced by a pressure difference between the entry and the output of the channel. The system will be stationary. The boundary conditions of the biomimetic surface are the no-slip boundary condition at the solid-water and solid-air interface, and shear force and velocity continuity at the air–water interface.

The mathematical schemes that define the above-mentioned conditions are described below. Considering a fully developed flow, where the fluid moves parallel to the microchannels (infinitely long channel on x), the velocity field is defined by $u=f\left(y,z\right)$. Because of this,

(1) $$\frac{\partial u}{\partial x}=\frac{\partial v}{\partial x}=\frac{\partial w}{\partial x}=0$$

In the case of incompressible flows, the continuity equation is reduced to:

(2) ∇⋅V=0

V is the velocity field of the fluid. As the flow has a laminar regime parallel to the microchannels, no secondary currents are induced. Then, v=w=0. Momentum equations will be:

(3) $$\rho (u\frac{du}{dx}+v\frac{\partial u}{\partial y}+w\frac{\partial u}{\partial z})=-\frac{\partial p}{\partial x}+\mu (\frac{\partial^{2}u}{\partial x^{2}}+\frac{\partial^{2}u}{\partial y^{2}}+\frac{\partial^{2}u}{\partial z^{2}})$$

(4) $$\rho (u\frac{dv}{dx}+v\frac{\partial v}{\partial y}+w\frac{\partial v}{\partial z})=-\frac{\partial p}{\partial y}+\mu (\frac{\partial^{2}v}{\partial x^{2}}+\frac{\partial^{2}v}{\partial y^{2}}+\frac{\partial^{2}v}{\partial z^{2}})$$

(5) $$\rho (u\frac{dw}{dx}+v\frac{\partial w}{\partial y}+w\frac{\partial w}{\partial z})=-\frac{\partial p}{\partial z}+\mu (\frac{\partial^{2}w}{\partial x^{2}}+\frac{\partial^{2}w}{\partial y^{2}}+\frac{\partial^{2}w}{\partial z^{2}})$$

And the conditions of the problem reduce these equations to:

(6) $$0=-\frac{\partial p}{\partial x}+\mu (\frac{\partial^{2}u}{\partial y^{2}}+\frac{\partial^{2}u}{\partial z^{2}})$$

(7) $$0=-\frac{\partial p}{\partial y}$$

(8) $$0=-\frac{\partial p}{\partial z}$$

The presented equations are valid for both fluids (water and air). The boundary conditions presented before can be written as:

No-slip for the fluid–polymer interface

For the top wall:

(9) z=H,∀x,y→u=v=w=0

For the top of the microchannel that is in contact with water:

(10) z=h;W≤y≤W+d→u=v=w=0 

The model will only consider one microchannel (0≤y≤W+d) (as the behavior will be the same for n microchannels), so there will not be any lateral walls. The boundary conditions for the sides of the channel will be:

(11) $$y=0;y=W+d;h\leq z\leq H\to \frac{\partial u}{\partial y}=0\;$$

In the case of the air trapped inside the microchannels,

(12) y=0,0≤z≤h;y=W,0≤z≤h;\breakz=0,0≤y≤W→u=0

Shear force and velocity continuity for the air–water interface

If the interface remains horizontal,

(13) $$z=h;\;0\leq y\leq W\to u_{air}=u_{water}$$

(14) $$z=h;\;0\leq y\leq W\to \mu_{air}\frac{\partial u_{air}}{\partial z}=-\mu_{water}\frac{\partial u_{water}}{\partial z}$$

Experiments will be performed within the laminar range for the regimen flow, as turbulent regimes are known for eliminating the air pockets that the superhydrophobic surfaces can provide. For this, a range of pressure gradients from 100 to 10,000Pa/m will be applied to the channel. Reynolds number will be calculated as:

(15) $$Re=\frac{4R_{H}u}{\upsilon }$$

Here, $R_{H}$ is the hydraulic radius, u is the average velocity within the channel and υ is the kinematic viscosity of the fluid (water). The friction factor will be calculated as:

(16) $$f=-\frac{\partial P}{\partial x}\frac{4H}{\rho u^{2}}$$

Here, $\frac{\partial P}{\partial x}$ is the pressure gradient applied to the system, H is the height of the channel, and ρ is the density of the fluid (water).

### 2.2. 3D printing of the microchannels

The microchannels were designed using the software Autocad (v. 2021), considering the characteristic dimensions and morphology of the rice leaf, which contains riblets having a height of 50µm, a width of 100µm, and a separation of 200µm (Figure 1b) [2]. Flat samples were also designed to compare the effect of the hierarchical structure.

For the manufacturing of the biomimetic surfaces, a Form 2 SLA 3D printer (Formlabs, USA) was used. This printer uses a 405nm laser beam to solidify a photocurable resin layer-by-layer, based on a computational design. The base material is a polymeric photocurable resin-type Clear of the brand Formlab, which allows for the highest resolution (25µm). According to the manufacturer information, its components are (%w/w): urethane dimethacrylate (55–70), methacrylate monomers (15–25) and a photoinitiator (<.9) [52]. Isopropyl alcohol (technical grade, Avalco, Chile) was used to wash and clean the printed polymeric surfaces.

### 2.3. Surface modification with TiO_2_ nanoparticles

The hydrophobic coating is prepared according to Zhang et al. [30]. Commercial TiO_2_ nanoparticles with a bimodal size distribution are used to favor the hierarchical structure (<22 and <100nm, according to the datasheet from Sigma-Aldrich, China, and Germany), as this kind of distribution of nanoparticle sizes allows a rough and robust coating [30]. Their surface wettability was modified using HTMS (technical grade, Sigma-Aldrich, Germany), with absolute ethanol as a solvent (p.a., Merck, Germany) (Figure 1c). In this reaction, HTMS hydrophobic chains are covalently bonded to the nanoparticles’ surface through the condensation reaction between methoxysilane moieties and the OH groups placed at the surface of the nanoparticles, turning the nanoparticles hydrophobic. The steps to produce the coating are as follows: (i) mixing 1.58mL of HTMS with 201.6ml of absolute ethanol at room temperature (RT), (ii) adding 8.4g of TiO_2_ nanoparticles (22nm), and magnetically stirring the solution for 2hours, (iii) addition of 8.4g of TiO_2_ nanoparticles (100nm) and magnetically stirring the solution for .5hours, followed by (iv) ultrasonication for 2.5hours.

Dip coating was used to coat the 3D printed structures, where the material is submerged for 2seconds one time (a single layer). After this, the sample is dried in a vacuum stove at 60°C for one hour. Then, the sample is washed under ultrasonic irradiation with ethanol (technical grade, Avalco, Chile) as a solvent for 5 min. The surface is then dried on the stove for 15 min, and then the dip-coating process is repeated (two layers). One of the advantages of this technique is that the coating can be retouched as many times as needed. Due to photocatalytic reactions reported for TiO_2_ nanoparticles [37], samples must be stored in a dark environment.

### 2.4. Chemical and topographical characterization

X-ray diffraction spectroscopy (×RD) spectra of the modified nanoparticles were obtained using a Bragg-Brentano powder X-Ray Diffractometer (model D8 Advance, Bruker), having a linear LynxEye detector (40 kV/30mA). Transmission electron microscopy (TEM) images of the nanoparticles were acquired from a Hitachi HT7700 microscope (120 kV). The thermogravimetric analysis (TGA) was performed between 28°C and 700°C at 10 °C/min under a nitrogen atmosphere in a NETZSCH TG 209 F1 Libra® instrument (Germany). Surface modification of the TiO2 nanoparticles was studied through attenuated total reflection Fourier-transform infrared spectroscopy (ATR-FTIR). ATR-FTIR spectra were acquired from a Thermo Scientific Nicolet iS 10 spectrophotometer coupled to an ATR Smart iTX accessory with a monolithic diamond crystal. The surface topography was observed using a field emission scanning electron microscope (FE-SEM). FE-SEM images were acquired with an FEI QuantaTM 250 microscope equipped with an Octane silicon drift detector for elemental analysis via energy-dispersive X-ray spectroscopy (EDS).

### 2.5. Contact angle measurements

Distilled water was used for the contact angle measurements using a Drop Shape Analyzer DSA25 goniometer (Krüss, Germany). These were performed using dynamic measurements (advancing and receding angle), as it becomes impossible to place a still drop over a superhydrophobic surface without gravity affecting its shape [53]. The drops were filled until they reached a volume of 10µL. Two measurements on each direction (perpendicular/parallel views to the microchannels, Figure 1d) were made per sample, three samples were analyzed per type of surface, and the error values presented correspond to standard deviation. The presence of the Cassie-Baxter state for each case was observed from the goniometer’s optical images.

Due to the inherent directionality of the printing filaments, the printed surface is anisotropic independent of the design used. To characterize this effect the advancing and receding contact angles were measured for the parallel and perpendicular orientation (Figure 1d). The first is measured by adding volume to the droplet, while the latter is the contact angle upon volume reduction [53]. Anisotropy was quantified by the difference between the perpendicular and parallel values [18,23], while hysteresis was characterized by the difference between the advancing and receding contact angles for the same orientation. Hysteresis measures the adhesion of a liquid droplet to the surface (or resistance to its remotion) as the pinning force is known to be proportional to the contact angle hysteresis [54], and a value <10° is required for superhydrophobic surfaces.

Dynamic experiments for drops velocity anisotropy and drops bouncing dynamics were recorded using a Chronos 1.4 high-speed camera (Kron Technologies, Canada).

## 3. Results and discussion

### 3.1. Numerical results

As discussed above, superhydrophobic surfaces enable the existence of air pockets between a drop of water and the surface arising from their hierarchical structure [17,55]. To confirm the effects that a rice leaf biomimetic surface with trapped air could have on the dynamics of water transport, we carried out numerical simulations of a simplified system consisting of water moving inside a rectangular channel under laminar flow conditions (that favor the existence of the air pockets) [56]. The superhydrophobic surface was simulated through a simplified structure consisting of a flat hydrophilic surface on the top wall and a single superhydrophilic microchannel surface at the bottom formed by the presence of a solid-riblet (right-side) and air pocket (left-side). The boundary conditions at the bottom surface are zero velocity at the solid-water interface as well as shear force and velocity continuity at the air–water interface. The lateral walls of the rectangular channels presented periodic boundary conditions to replicate the periodical arrangement of the experimental biomimetic surface. Both channels have the same height of H=9.5 · 10^−4^ m (not including the microchannel), the channel width is 2 · 10^−4^ m, the microchannel width is 1 · 10^−4^ m and its height is 5 · 10^−5^ m. We aim at comparing the velocity profiles obtained from this superhydrophobic surface having microchannels with the results from a flat surface. The simulations were conducted for pressure gradients of 100, 500, 1000, 5000 and 10,000Pa m^−1^ to allow a laminar flow under the specific channel dimensions.

**Figure 2.**
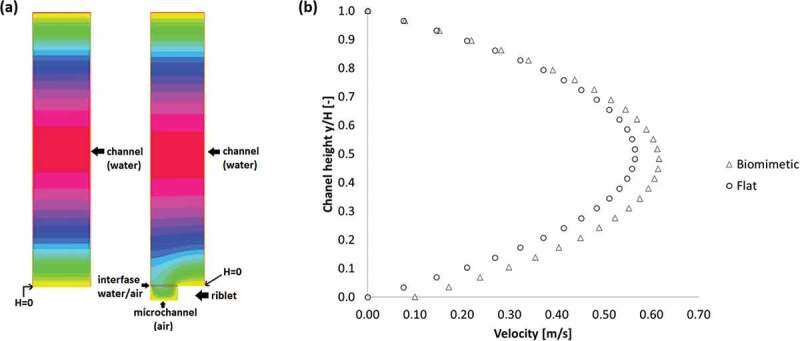
(A) Velocity profiles obtained via numerical simulations for a pressure gradient of 5000 Pa m ^−1^ on a flat channel (left) and a surface with one microchannel at the bottom wall (right). Maximum velocities are at the center of the channel (red): .58m s ^−1^ and .63m s ^−1^, respectively. No-slip boundary conditions can be observed at the solid–fluid interface (yellow), and velocity continuity (slip velocity) can be observed at the air-water interface (microchannel, red line). (b) Velocity profiles were obtained via numerical simulations for the flat channel and the surface with one microchannel for a pressure gradient of 5000 Pa m ^−1^. the plotted velocity value corresponds to the average velocity for each channel height.

An example of the velocity profiles obtained for each configuration of the channel (flat and with microchannel) is displayed in Figure 2(a,b) for a pressure gradient of 5000Pa m^−1^. In this context, the effect of the microchannel at the bottom wall can be approximated as a change in the shape of the velocity profile (Figure 2b), as a non-zero value at the biomimetic surface (H=0) increasing the maximum velocity at the center of the channel by 9%, and the average velocity by 13%. This phenomenon is known as ‘slip velocity’ [57] and occurs when the shear force exhorted by the surface (air/water interface) over the fluid is not enough to stop the layer that advances at the interface (boundary layer), which can be theoretically modeled as a penetration of the boundary layer into the material [58]. For superhydrophobic surfaces, this slip velocity is generated through the decrease in the shear force exerted by the air pockets compared to the solid surface due to its small viscosity compared to water [59]. The values obtained for the average velocity, slip velocity, shear stress, and friction factor for each case are presented in Tables 1 and 2 (for the flat and biomimetic bottom walls, respectively).

**Table 1.** Numerical simulations results for the flat bottom wall

Flat bottom wall					
Pressure gradient [Pa/m]	Re	Average velocity [m/s]	Slip velocity [m/s]	τ bottom wall [N/m^2^]	Friction factor *f* [-]
100	14	7.53×10^−3^	0	4.36×10^−5^	6.16×10^−3^
500	72	3.77×10^−2^	0	2.18×10^−4^	1.23×10^−3^
1000	143	7.53×10^−2^	0	4.36×10^−4^	6.16×10^−4^
5000	715	3.77×10^−1^	0	2.18×10^−3^	1.23×10^−4^
10000	1431	7.53×10^−1^	0	4.36×10^−3^	6.16×10^−5^

**Table 2.** Numerical simulations results for the biomimetic bottom wall

Biomimetic bottom wall					
Pressure gradient [Pa/m]	Re	Average velocity [m/s]	Slip velocity [m/s]	τ bottom wall [N/m^2^]	Friction factor *f* [-]
100	22	8.53×10^−3^	2.01×10^−3^	4.12×10^−5^	4.54×10^−3^
500	108	4.26×10^−2^	1.00×10^−2^	2.06×10^−4^	9.09×10^−4^
1000	216	8.53×10^−2^	2.01×10^−2^	4.12×10^−4^	4.54×10^−4^
5000	1080	4.26×10^−1^	1.00×10^−1^	2.06×10^−3^	9.09×10^−5^
10000	2160	8.53×10^−1^	2.01×10^−1^	4.12×10^−3^	4.54×10^−5^

The microchannel surface allows for a 26% reduction of the friction factor compared to the flat channel. Under these conditions, the Re values also increased proportional to the velocity increase, although showing values lower than 2200 meaning laminar flow in all the cases (controlled by the pressure gradients). The slip velocity has a direct proportionality with the pressure gradient, and its value is about 24% of the average velocity on the experiments with microchannels. Flat channels do not provide a slip velocity, as a no-slip boundary condition was imposed for this type of surface. At higher pressure gradients (displaying turbulent flow), the air pockets formed by the biomimetic surface are not present [56]. The simulated slip length provided by the biomimetic configuration can be estimated by extending the velocity profile slope obtained at the surface/water interface until reaching the position where the boundary layer would theoretically end [60], obtaining a value of b=8.96×10^−5^ m. This corresponds to 10% of the total height of the channel, which could allow for 23% of drag reduction in a Poiseuille flow configuration according to Choi et al. [61].

Considering the effect of a biomimetic wall in the simulations carried out, it is expected that the experimental results show a relevant change in the interaction of water droplets with the different surfaces given the presence of air gaps, decreasing the adhesion of the material with the fluid. This effect will be reflected in the advancing and receding contact angle measurements, where less friction of the material with the water will deform the droplets interacting with the surface less. The speed of the droplets, when deposited on inclined surfaces, should increase considerably.

### 3.2. Surface characterization

#### 3.2.1. Analysis of TiO_2_-HTMS nanoparticles and coated surface

**Figure 3.**
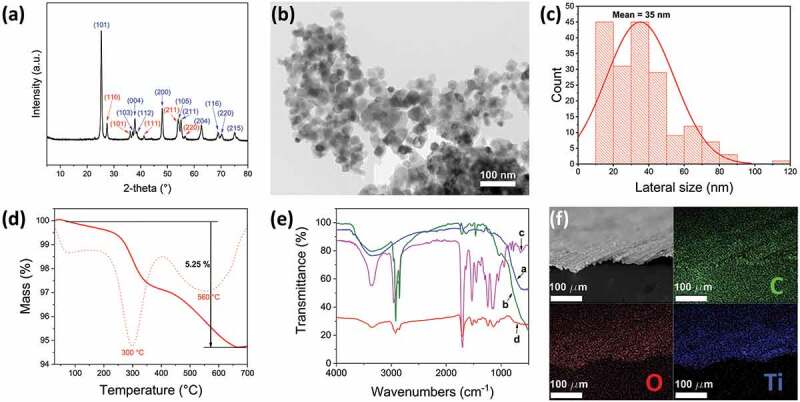
Microstructural and chemical characterization of TiO_2_-HTMS nanoparticles and coated surface. (a) Powder XRD diffractogram of TiO_2_-HTMS nanoparticles (blue and red indices correspond to anatase and rutile phases, respectively). (b) Representative TEM image of TiO_2_-HTMS nanoparticles. (c) Histogram of TiO_2_-HTMS nanoparticle’s size distribution. (d) TGA curve of TiO_2_-HTMS nanoparticles under a nitrogen flow (dotted line corresponds to the first derivative of the TGA curve). (e) ATR-FTIR spectra of TiO_2_ (blue line, a), TiO_2_-HTMS (green line, b), pure 3D printed resin (magenta line, c), and TiO_2_-HTMS coated 3D printed resin (red line, d). (f) SEM-EDS elemental analysis for the surface of the TiO_2_-HTMS coated polymeric resin (top left) showing the presence of carbon (green), oxygen (red), and titanium (blue).

The modified nanoparticles (TiO_2_-HTMS) were characterized through XRD and TEM techniques. Figure 3a shows the powder XRD diffractogram of TiO_2_-HTMS nanoparticles presenting diffractions peaks at 2θ=25.3 °, 37.0 °, 37.9 °, 38.5 °, 48.0 °, 54.0 °, 55.1 °, 62.7 °, 68.9 °, 70.3 °, 75.2 °, and 2θ=27.5 °, 36.1 °, 41.3 °, 53.8 °, and 56.6 °, corresponding to mixed phases of anatase and rutile, respectively [62,63]. TiO_2_-HTMS nanoparticles exhibit compact self-organization, as shown in the TEM image of Figure 3b arising from the attractive interaction of the alkyl chains on the surface of the nanoparticles. The size of the spherical nanoparticles is shown in Figure 3c as a distribution histogram. The mean size of TiO_2_-HTMS is 35nm, exhibiting a high frequency of nanoparticle sizes between 10–20nm and 35–40nm. The chemical modification of nanoparticles does not change the commercial product’s size. Figure 3b otherwise shows well disperse nanoparticles meaning a posterior efficient and well-packed coverage.

TGA estimated the degree of functionalization of TiO2-HTMS nanoparticles. Thermal degradation of silanized metal oxide nanoparticles provides information of the organic content [64]. Figure 3d shows the thermogram of TiO_2_-HTMS under a nitrogen atmosphere. The curve exhibits a 5.25% mass loss between 100°C and 650°C corresponding to the organic HTMS moiety. The mass loss in two steps could be due to the different sizes of nanoparticles, each one having a different modification degree due to the different surface area. The chemical modification of TiO_2_ nanoparticles and the coating of the 3D printed structures were studied via ATR-FTIR spectroscopy (Figure 3).

Figure 3e shows the FTIR spectra of the nanoparticles before and after silanization with HTMS as well as the 3D printed resin before and after the application of the hydrophobic coating. The spectrum of TiO_2_ (blue line in Figure 3a) shows a broadband at 3350cm^−1^, corresponding to the stretching vibration mode of the O-H bonds of hydroxyl groups, a weak band at 1630cm^−1^, corresponding to the bending vibration mode of the O-H bonds of moisture water, and an intense band at 600cm^−1^ associated to stretching vibration mode of the Ti-O-Ti bonds [65,66]. The spectrum of TiO_2_-HTMS (green line in

Figure 3a displays two new sharp and medium intensity bands at 2920cm^−1^ and 2850cm^−1^, corresponding to the asymmetrical and symmetrical stretching of the C_sp3_-H bonds of alkyl chains [67]. It also shows bands around 1465cm^−1^, 1170cm^−1,^ and 1030cm^−1^, corresponding to scissoring of C_sp3_-H bonds, and symmetrical as well as asymmetrical stretching of Si-O-Si bond (formed by condensation), respectively [68]. The shoulder of the band observed in the TiO_2_-HTMS spectrum at 950cm^−1^ corresponds to vibrational modes of Ti-O-Si bonds [69], which implies that the nanoparticles were successfully modified by HTMS.

The commercial photo-cross-linkable resin used for the 3D printing is based on urethane dimethacrylate and other methacrylate monomers [52]. This resin presents bands at 3350cm^−1^, 2950cm^−1^, 2860cm^−1^, 1700cm^−1^, 1530cm^−1^, 1452cm^−1^, 1240cm^−1^ and 1150cm^−1^, corresponding to the respective vibrational mode of *N*-H (stretching), C_sp3_-H (asymmetrical stretching), C_sp3_-H (symmetrical stretching), C=O (stretching), C-N (stretching) combined with *N*-H (bending), C_sp3_-H (bending), C-O (ester, stretching) and C-O-C (stretching) bonds (magenta line in Figure 3a), respectively [70]. After coating the 3D printed structure with TiO_2_-HTMS (red line in Figure 3e), the characteristic bands of TiO_2_-HTMS and the polymer substrate were observed, confirming the physical bonding of the modified nanoparticles on the 3D printed structure. FE-SEM image with elemental analysis results (Figure 3f) shows two of the elemental components of the commercial resin (C and O), and further confirms the effective coating of the 3D printed structures by dip coating (Ti).

#### 3.2.2. Surface topography

**Figure 4.**
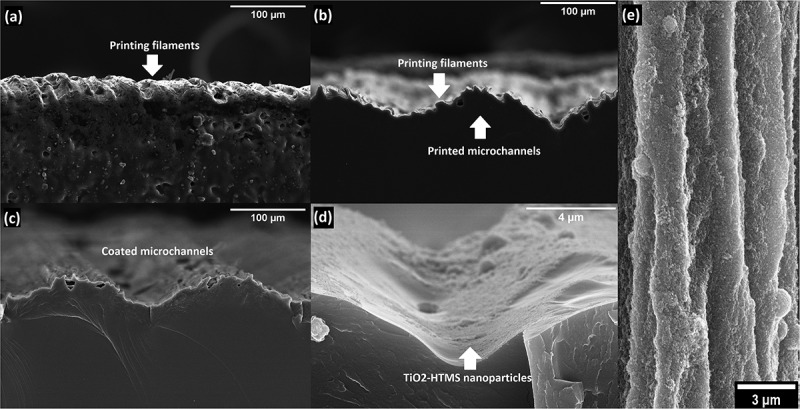
FE-SEM images of the 3D SLA printed surfaces (perpendicular view, plane zy): (a) flat surface showing printing layers, (b) printed microchannels (100µm designed height) with printing layers on top, (c) biomimetic coated microchannels, (d) TiO_2_-HTMS nanoparticles deposited on the microchannels, and (e) top view of the TiO_2_-HTMS nanoparticles placed over the printing filaments.

FE-SEM images of the flat and the micropatterned printed surfaces are displayed in Figure 4. The flat surface (Figure 4a) displays a roughness of around 10µm that is characteristic of the printing filament used for SLA 3D printing. This roughness implies that the printed surface is anisotropic depending on the printing direction. The surface topography of the printed microchannels shows rounded edges (Figure 4b) instead of the designed perfect rectangles due to the spot-size of the laser beam (around 140µm) used [71], as the microchannels are printed perpendicular to the printing layer orientation (the printing layers are added in the plane zy, adding height on the axis *x* of Figure 1b). However, this mismatch between the designed and obtained morphologies even amplifies the biomimetic aspect of the surface as rice leaves have wavy riblets. The printed channels further mismatch the designed dimensions, especially their height (from 50 to 30µm). To overcome this limitation, three structures with different heights (50, 75, and 100µm) were 3D printed allowing for further analysis of the effect of this variable. Noteworthy, the roughness arising from the 3D printed filaments (observed in the flat surface) is also displayed in this case providing a hierarchical roughness to the microchannels thus improving the similarity to the papillae observed on the rice leaf surface (Figure 1a). After applying TiO2–HTMS coating with two particle sizes (22 and 100nm) (Figure 4c), the nanoparticles are distributed over the printing filaments (Figure 4(d,e)). Figure 3b shows a TEM image of the nanoparticles used to coat the surface, where the different nanoparticle sizes are arranged randomly and close-packed over the treated surface (Figure 4e).

**Figure 5.**
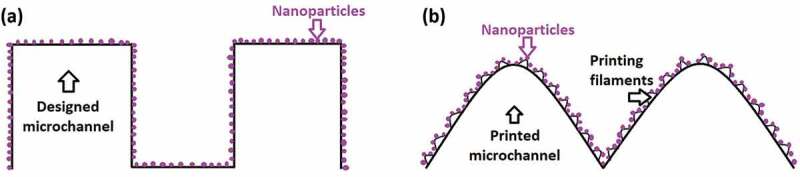
(A) Designed two-level hierarchical surface for the biomimetic material: designed microchannel and nanoparticles. (b) Actual three-level hierarchical surface for the biomimetic material: printed microchannel, printing filament, and nanoparticles.

These results show that the surface coating of an SLA 3D printing material mismatches the original rectangular design (Figure 5a) and allows for the generation of a three-level hierarchical structure biomimicking the morphology of the rice leaf surface associated with: 3D printed microchannels (100µm scale), roughness from the printing filaments (10µm scale), and nanoparticles (22 and 100nm scale), as displayed in Figure 5b.

3D printing has been previously used for developing biomimetic structures and characterized regarding the roughness. For instance, the characteristic roughness of the SLA 3D printing technique is also reported in the work of Wang et al. [72] for developing hydrophobic surfaces, where structures of micrometric posts (diameter of 300µm) are manufactured and roughness of the order of 30µm is obtained on their sides. However, by using this configuration the printing filaments do not interact with a drop of water, since they are not present on the upper face of the posts and do not necessarily contribute to the wettability of the surface unless the drop penetrates the air spaces (where roughness could even increase the adhesion of the fluid). Li et al. [40] used the multiscale SLA 3D printing technique to produce a lotus flower surface and shark denticles but did not report a characteristic roughness for the printing filaments. 3D printing by TPP (two-photon polymerization) is another technique that has been used to produce biomimetic microstructures [73] inspired by the surface of the *Salvinia molesta* plant. Although they managed to print eggbeater-like structures of the order of 10µm, their surfaces are smooth and without roughness on a smaller scale, which is observed in the natural sample of the plant. The *Salvinia molesta* surface has also been mimicked using immersed surface accumulation 3D printing [28]. Likewise, 3D printing by FDM (fused deposition modeling) has been used to manufacture biomimetic structures of the lotus flower [74] and others similar to the rice leaf [23]; however, none of these works report a hierarchical roughness attributable to the 3D printing technique.

#### 3.2.3. Surface wettability

**Figure 6.**
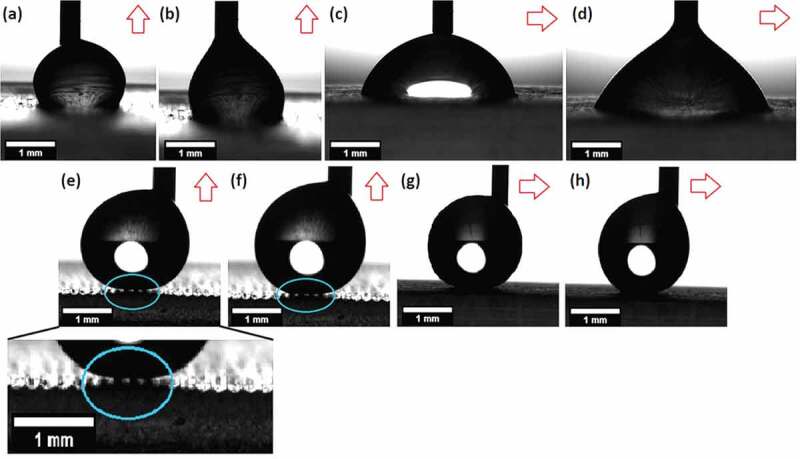
Optical images of the wettability of the flat uncoated surface: (a) perpendicular advancing, (b) perpendicular receding, (c) parallel advancing, and (d) parallel receding; and biomimetic surface (100µm coated microchannels): (e) perpendicular advancing, (f) perpendicular receding, (g) parallel advancing, and (h) parallel receding. Trapped air in the biomimetic surface can be observed in images (e) and (f) in the blue circle. the printed microchannel direction (view) is indicated by a red arrow (↑ perpendicular and → parallel).

Examples of the contact angles measured for the flat and the coated biomimetic surfaces are shown in Figure 6 for the advancing (Figure 6(a,e)) and receding (Figure 6(b,f) contact angle in the perpendicular direction as well as the advancing (Figure 6(c,g)) and receding (Figure 6(d,h)) contact angles in the parallel direction, respectively. By measuring these values, two relevant parameters were measured: anisotropy and hysteresis.

- Uncoated surfaces

**Figure 7.**
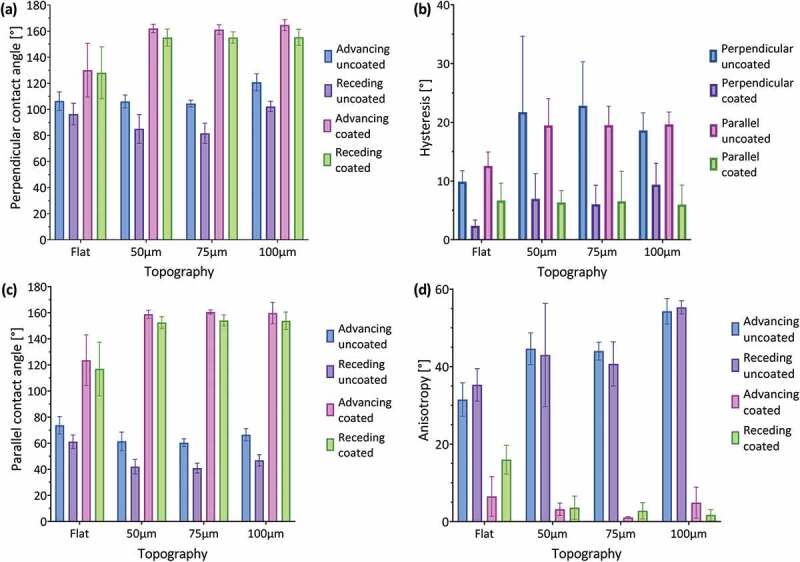
(A) Contact angle measurements (advancing and receding) perpendicular to the printing filaments, (b) contact angle hysteresis measurements for the perpendicular and parallel orientations, (c) parallel measurements of the contact angle (advancing and receding), and (d) contact angle anisotropy (difference between the perpendicular and parallel direction measurements for the advancing and receding contact angle) for the flat surfaces and the ones with microchannels (50, 75, and 100µm), with and without TiO_2_-HTMS coating.

The contact angles obtained for the perpendicular orientation are summarized in Figure 7a. The flat surfaces display values of 106±7° and 96±8° for the advancing and receding contact angle, respectively, with an average hysteresis value of 10°. The hydrophobic behavior observed for the flat uncoated surfaces can be explained through the chemical composition of the polymeric resin main component (urethane dimethacrylate), which structure is mainly apolar.

Surfaces having microchannels with a depth of 50 and 75µm maintain an advancing contact angle of around 106±5°, but these samples decrease the receding contact angle to 85±11° and 82±8°, respectively, thus increasing the hysteresis to around 21° (Figure 7b). Surfaces with 100µm microchannels exhibit an increased advancing and receding contact angle of about 121±7° and 102±4°, respectively, which implies a hysteresis of 18°. Hysteresis results from the higher adhesion of a drop during its volume reduction or receding process (Figure 6b) and contact angle measurements show that the topography increases this adhesion thus duplicating the hysteresis (~20°) as compared with the flat samples (10°). All uncoated samples (including the flat surface) show advancing contact angles associated with a hydrophobic wettability (>90°), with microchannels of 100µm presenting the highest hydrophobicity (15° higher values compared to the flat sample) thus confirming the relevance of the topography. Receding contact angles behave hydrophobically for the flat surface and microchannels of 100µm (96 ° and 102 °, respectively), while the surfaces with microchannels of 50 and 75µm presented hydrophilic values <90°.

The contact angles for the parallel orientation are presented in Figure 7c shows lower values (less hydrophobic) compared to the perpendicular measurements for all uncoated samples. In this case, the drop of water does not have to move through changing topography during the dynamic tests, meanwhile, for the perpendicular tests, the drop must move alternately between the peaks and valleys of the riblets. For instance, for all uncoated surfaces, the advancing contact angles have values lower than 74±7°, while the receding contact angles decreased to values lower than 60±5°. Despite these changes, hysteresis values for both orientations are similar in the case of the uncoated surfaces (Figure 7b). This implies that the pinning force between the material and the fluid does not depend on the orientation of observation.

The results of the anisotropy measurements are presented in Figure 7d. The flat surface shows a difference of 32±4° and 35±4° between perpendicular and parallel measurements for the advancing and receding contact angle, respectively. This confirms that the printing filaments create a slightly anisotropic surface, as can be seen in Figure 6(a–d). This behavior is amplified by the presence of microchannels, which increase the anisotropy to values around 45±4° for the advancing and receding angles (50 and 75µm microchannels), and 55±4° (100µm microchannels), respectively. This tendency results from the higher perpendicular contact angles obtained for increased structural depths while keeping the contact angle constant in the parallel direction across all depths fabricated.

- Coated surfaces

Perpendicular measurements show that all coated surfaces become more hydrophobic as compared to the uncoated surfaces (Figure 7a). The flat-coated surface increases its advancing contact angle to 130±21° (25° higher than uncoated surface), and all microchannels presented angles higher than 161±4°, which implies a superhydrophobic behavior, surpassing the values obtained by previous studies [2,8,23]. In the case of the microchannels with a depth of 100µm, an average value of 165±4° was obtained, which coincides with the characteristic value of 164° of the actual rice leaves. The receding contact angle presented a similar tendency increasing to 128±20° for the flat-coated surface and to values higher than 155±7° for all microchannels. The hydrophobic coating decreased the hysteresis values (Figure 7b) to 2° and lower than 9° for the flat and microchannel surfaces, respectively, which is a further requirement for superhydrophobic surfaces (<10°) [15]. Pictures from these tests show that air pockets exist at the interface between the drop and the surface explaining this superhydrophobic behavior through a Cassie-Baxter state (Figure 6e).

The advancing contact angle in parallel orientation (Figure 7c) also increased significantly after coating to 124±20° for the flat surface (50° higher) and to 160±8° (100° higher) for all microchannels. The receding contact angle values increased to 117±21° for the flat surface (57° higher) and to >153±4° for all microchannels (at least 113° higher). These higher differences compared to the perpendicular values mean that the coating significantly decreases the adhesion of the fluid to the surface in the parallel direction. Hysteresis values (Figure 7b) are lower than 7° for all coated surfaces and do not show significant differences compared to the perpendicular measurements.

Anisotropy measurements (Figure 7d) show that the coating has a pronounced effect on all surfaces. Flat samples reduce their anisotropy values to 7±5° and 16±4° for the advancing and receding contact angle, respectively (over 20° difference compared to the uncoated case). Microchannels having a depth of 50 and 75µm decrease their anisotropy values to <4±2° (compared to 45° for the uncoated case), while 100µm microchannel with a depth of 100µm show values of 5±4° and 2±1° for the advancing and receding contact angle, respectively (compared to 55° for the uncoated case). This tendency coincides with the behavior of both a natural rice leaf with an advancing contact angle anisotropy of 6° and previous biomimetic rice leaf surfaces developed via mold imprinting coated with silica nanoparticles (5°) [2]. The decreased anisotropy after coating confirms the presence of air pockets at the interface that avoid the direct interaction between the fluid and the surface.

**Figure 8.**
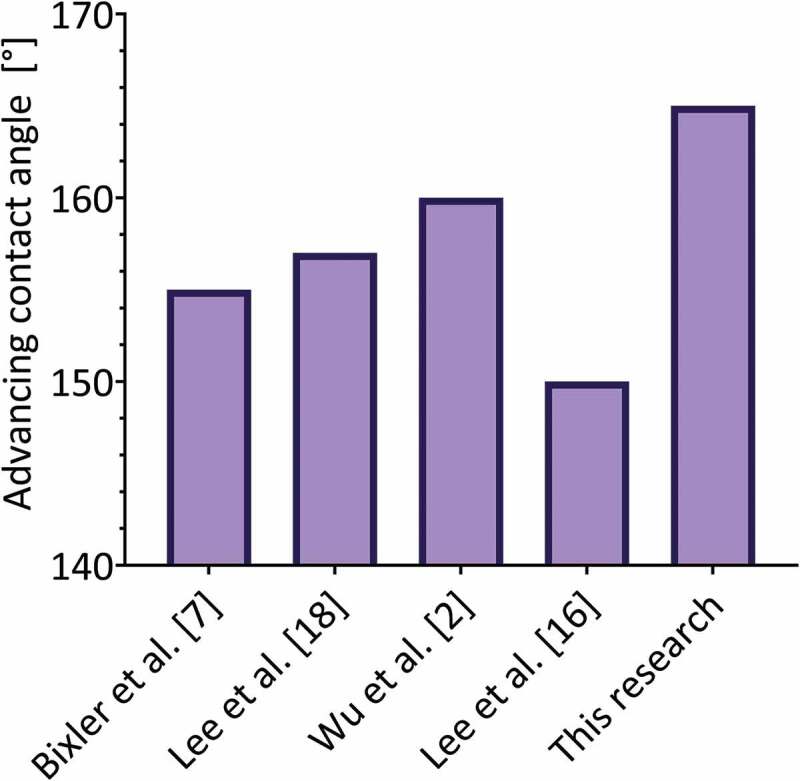
Comparison between the advancing contact angle (perpendicular direction) obtained for previous investigations and this research.

Regarding previous investigations, Figure 8 shows the comparison between the advancing contact angle measurements obtained for Bixler et al. [8], Lee et al. [23], Wu et al. [2], Lee et al. [18], and this research. Bixler et al. [8] report a contact angle of 155 ° for a polymeric urethane surface molded with a natural sample of the rice leaf and coated with silica nanoparticles, which implies an increase of 37 ° compared to the uncoated sample. This increase is less than the one obtained for the material developed in this work, which corresponds to 44 ° for the surface with 100µm microchannels. Regarding the hysteresis of the contact angle, the authors report around 8 ° for the coated biomimetic surface, a value very similar to the 9 ° obtained for the perpendicular orientation of the newly developed material. This indicates that both materials have a very similar adhesion to a drop of water, reflecting the great biomimetic aspect of the material developed in the present investigation compared to a surface developed with a mold of a natural rice leaf. However, the perpendicular contact angle obtained in this work exceeds the reference by 10 °.

Lee et al. [23] reported a contact angle of 114 ° for a smooth PLA material printed in 3D by FDM and coated with silica nanoparticles, which is less than the advancing contact angle measured on the flat-coated surface developed in this work both in the perpendicular and parallel direction (130 and 124 °, respectively). In the case of the surfaces presented by the authors with 100µm microchannels, they reported a perpendicular contact angle of 157 ° and parallel contact angle of 150 °, lower values than those reported in this work (165 and 160 °, respectively). Although the authors do not report hysteresis values for the contact angle, they do report the sliding angle, which corresponds to 12 ° for the perpendicular orientation and 10 ° for the orientation parallel to the microchannels. This does not coincide with the <5 ° limit required in the slip angle for superhydrophobic surfaces that do not adhere to water, therefore this material does not meet the requirements to be non-adherent and possible reducer of drag forces.

Wu et al. [2] developed biomimetic structures of the rice leaf using PDMS molds and silica nanoparticles, using different dimensions (10–60µm deep and 20–200µm wide). They report a contact angle of 160 ° (5 ° less than in this work) and measure the adhesion of the surface to a drop of water by the sliding angle. These surfaces, as they are not printed, do not show roughness due to the presence of printing filaments, and only show a hierarchical two-level structure (microchannel – nanoparticle). The authors indicate that the anisotropy of the sliding angle increases with the depth of the microchannels and the spacing between them, the latter being the most relevant parameter. All reported sliding angle values are <8 °, which implies that all configurations show low adhesion to a drop of water.

Lee et al. [18] reported micro-grooves 40µm wide (PDMS) coated with nano-silica, which depending on the number of coating layers can have a contact angle of 170 ° (RMS roughness 80nm), a value that can even be exceeded when the coating generates an isotropic surface (completely covers the microchannels). For the best anisotropic case (roughness RMS 80nm, 9 coating layers), the hysteresis of the contact angle has a value close to 10 °, thus fulfilling the requirement for a superhydrophobic material that does not adhere to a drop of water. However, for the case with 2 coating layers (comparable with the present work), which corresponds to an RMS between 20–40nm as indicated by the authors, the static contact angle barely reaches 150 ° for the perpendicular orientation (<150 ° for the parallel orientation, at least 15 ° less than in this work), and the authors indicate that the droplets adhere to the surface. According to this, the material presented in this work does not exceed the wettability properties granted by the hierarchical structure developed in the present investigation.

Therefore, superhydrophobic biomimetic materials can be designed through an SLA 3D printer and a nano-coating that is different than previous investigations that mimicked a rice leaf hierarchical structure surface and wettability using silica nanoparticles [2,8,23].

The contact angle results confirm that only the printing filaments, the printed microstructure, or the nanoparticle coating, cannot reach a superhydrophobic wettability by themselves or combine only two of them. Therefore, the three-level hierarchical structure must be present to obtain the desired superhydrophobic behavior. Hereafter, complementary experiments were carried out on the 3D printed biomimetic surface with the highest depth of 100µm resulting in the greatest superhydrophobicity.

### 3.3. Dynamic experiments

#### 3.3.1. Dynamics of water drops

**Figure 9.**
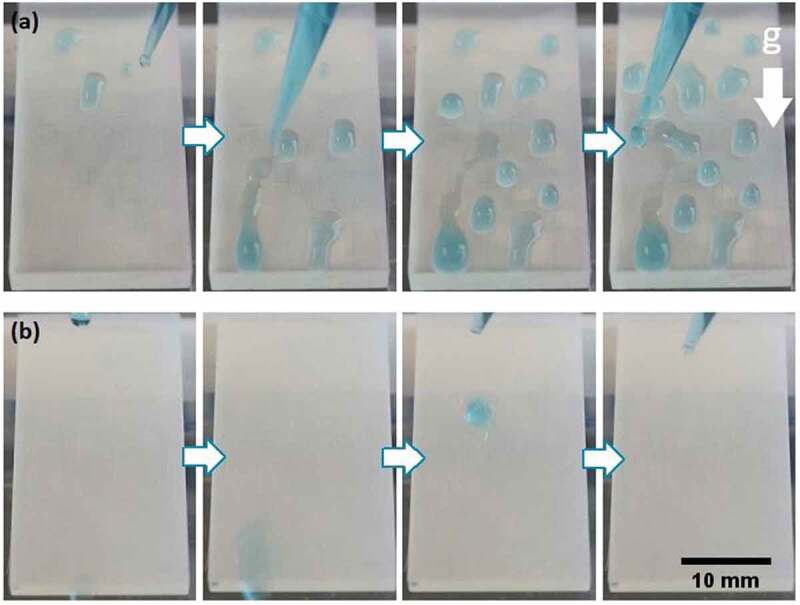
Dynamic adhesion experiments: (a) flat uncoated surface with inclination, water drops (blue) are placed over the material with a pipette (blue) and they adhere to it. (b) inclined biomimetic surface (100µm designed coated microchannels), a water drop (blue) is placed over the surface, and it rolls off without any resistance to motion induced by the material.

Dynamic experiments were conducted on an inclined surface (25°) to observe the movement of a water droplet (having methylene blue for visualization purposes) using both the uncoated 3D printed flat surface and the 3D printed biomimetic rice leaf material (Supporting Video 1). On the uncoated hydrophilic 3D printed flat surface, the water droplets spread and attach to the material (Figure 9a), while on the biomimetic surface the water droplets do not experience resistance to motion thus flowing over the material (Figure 9b).

This behavior agrees with the results from the contact angle measurements (Figure 7) as the presence of a three-level hierarchical structure formed by the printing filaments, 3D printed microchannels, and the nanoparticle coating renders superhydrophobic characteristics to the material due to the presence of air pockets at the interface.

**Figure 10.**
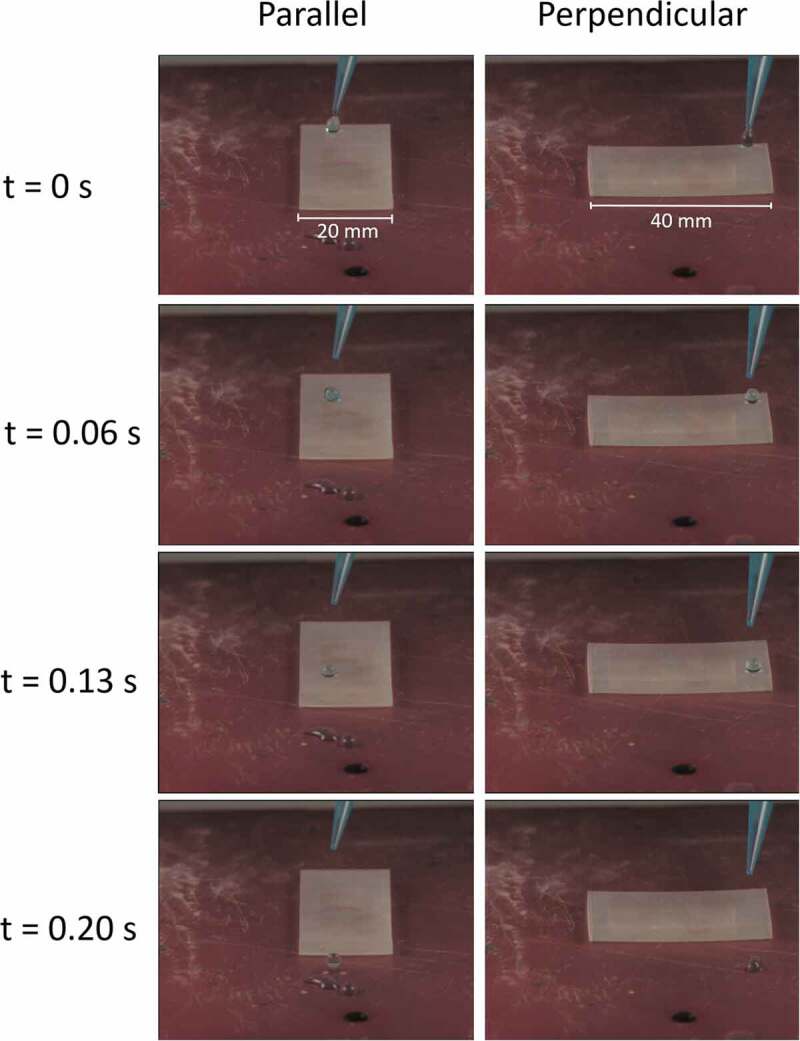
Drop velocity comparison for the biomimetic inclined surface (25°) between the parallel and perpendicular direction to the printed microchannels. the drops have a volume of approximately 14µl, the surface has a width of 20mm and a length of 40mm.

The anisotropy of the biomimetic surface was studied measuring the velocity of drops that roll over inclined surfaces, in the perpendicular and parallel direction to the printed microchannels (Figure 10, Supporting Video 2). Three different drops with an average volume of 14µL were placed over the inclined surface (25 °) in each direction, and then a video was recorded using a high-speed camera (2873 fps). Results show that drops have an average velocity of 239mm/s in the parallel direction, and 154mm/s in the perpendicular direction (55% higher in the parallel direction). This represents the natural behavior of water drops that roll over rice leaves, which prefer the parallel direction to the microchannels, as riblets act as a guide for the droplets to move over the surface.

#### 3.3.2. Self-Cleaning properties

**Figure 11.**
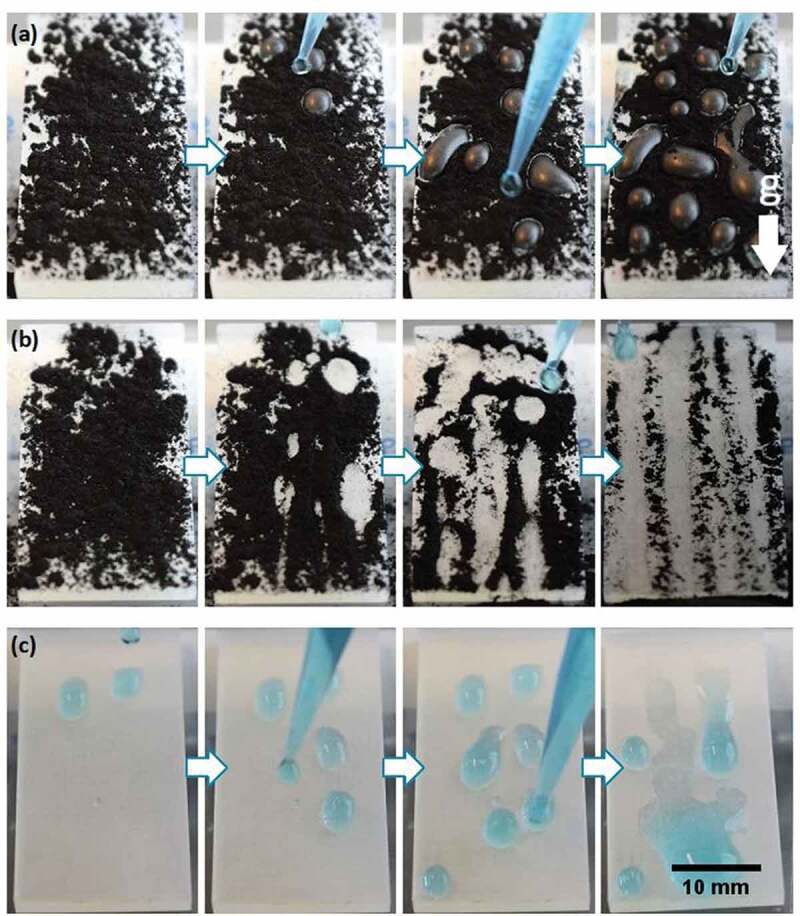
(A) Self-cleaning experiment for the flat printed surface inclined and covered with activated carbon and washed with distilled water. as the drops adhere to the surface, the contaminant cannot be removed. (b) Self-cleaning experiment for the biomimetic surface (100µm designed coated microchannels) inclined and covered with activated carbon. as the water drops do not adhere to the surface and roll off easily, they carry the contaminant away and clean the surface. (c) Dynamic adhesion experiment for an inclined biomimetic surface after 30minutes of UV light radiation, drops of water (blue) are placed over the surface and they adhere to the material.

One of the main properties arising from natural superhydrophobic surfaces is self-cleaning, which can be observed in leaves that remove pollution by a simple rain shower as water drops roll over the surface thus removing the contaminants [75]. In plants, hydrophobic self-cleaning surfaces may both protect against harmful microorganisms, which are growth-inhibited by dry plant surfaces and ensure efficient gas exchange by keeping a thin film of air clinging to the surface when the leaves are submerged [76]. This effect has been drawing attention in the last few years for different applications in materials science, such as fabrics, photovoltaic panels surfaces, anti-corrosion, and fog-harvesting surfaces, among others [77–79]. To test the self-cleaning behavior in our case, activated carbon powder was scattered over inclined surfaces (both flat and biomimetic 3D printed materials) mimicking organic contamination, and then water drops were added (Supporting Video 3) [32]. In the case of the uncoated inclined flat 3D reference surface (Figure 11a), the water drops adhered to the material. Although they adsorbed the carbon powder, the contaminant was not removed from the surface (even after the addition of 30 drops). In contrast, when water drops were located on the top of the inclined 3D printed biomimetic surfaces (Figure 11b), they rolled off removing the contaminant. The surface stayed with a very low amount of carbon after the addition of just 30 water drops, mimicking the self-cleaning characteristic of the rice leaf. This is due to the decreased shear force that the biomimetic surface exerts over the water drops allowing them to roll over it easily due to the air pockets existing between the material and the fluid (Figure 6e). This self-cleaning property has been previously reported on flat plates coated with TiO_2_ nanowires [32], PDMS biomimetic surfaces coated with silica nanoparticles [8], perfluorosilane-coated titanium dioxide nanoparticles sprays [33], and perfluorodecyltriethoxysilane-modified silica/PDMS sprays [80], all without the involvement of 3D printing, and on 3D multijet printed biomimetic feathers of the bird *Anser cygnoides domesticus* [81].

#### 3.3.3. Effect of UV-A irradiation

Previous investigations have shown that TiO_2_ coated surfaces can change their wettability when exposed to UV light irradiation going from superhydrophobic to hydrophilic behavior, because of the photocatalytic properties of TiO_2_ nanoparticles [32]. Our rice leaf biomimetic surface was placed under UV-A light radiation (365nm, 48 W) for 30minutes and then, the dynamic adhesion experiment was repeated (Figure 11c) showing a dramatic change in the surface behavior since the drops adhered to the surface after irradiation instead of freely rolling off (see Figure 9b).

This change from superhydrophobic to hydrophilic behavior can be explained through the photocatalytic properties of TiO_2_ molecules, which increase the -OH groups after UV-A irradiation [82]. These groups can react and easily attach water molecules, which explains the affinity of the surface for water droplets. The UV irradiation effect provides the material a tunable wettability and can increase the application possibilities for the new biomimetic surface. An example of this is mist collecting surfaces, which are characterized by combining superhydrophobic and hydrophilic areas to direct dew drops that settle on the surface, for which they stand out as a low-cost, accessible, and energy-saving mechanism, that provides a feasible alternative to obtain freshwater [83,84].

### 3.4. Floatability experiments

Floatability is observed in nature in the insect known as water strider (*Gerris remigis*), which can float and walk over water due to its superhydrophobic legs that trap air on its surface [85]. The trapped air enabling the superhydrophobic behavior also influences the dynamics of a submerged object. This was verified in a study presented by McHale et al. [86] in which the air plastron (i.e. trapped air) formed over the surface of submerged spheres allowed up to 10% of drag reduction, observed as an increment on their terminal velocity. Dong et al. tested the effect of a superhydrophobic surface on the velocity of model ships, obtaining 39% drag reduction compared to uncoated flat surfaces [87].

**Figure 12.**
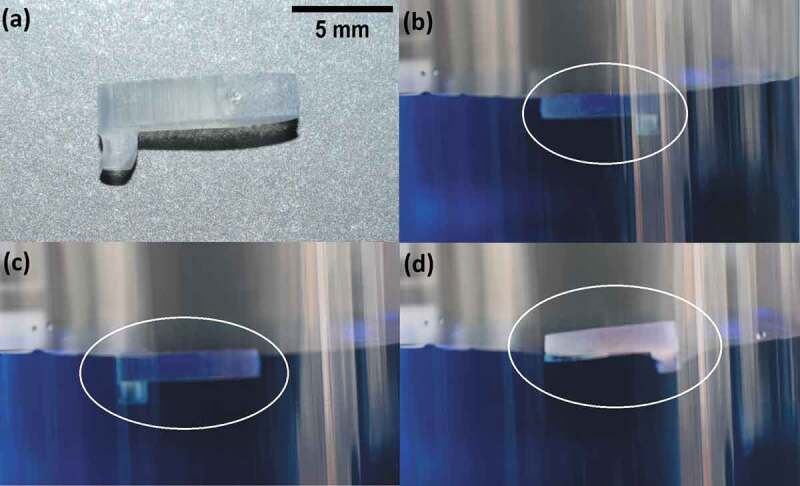
Floatability experiments for a small boat (a) on water (colorized with methylene blue) for an uncoated flat surface(b), 100µm microchannel uncoated surface (c), and a rice leaf biomimetic surface (100µm microchannel coated surface) (d). the position of the boat is indicated by a white circle. Trapped air can be observed at the keel of the biomimetic boat (d).

A small boat (Figure 12a) was 3D printed to confirm the drag reduction effect in our biomimetic superhydrophobic surface by floatability experiments as compared to a standard 3D printed surface. Three types of 3D printed surfaces were tested: flat uncoated, and both uncoated and coated 100µm microchannels. The boats, having a weight of .8 grams and measuring 10mm of length, 4mm of height, and 2mm thick, were gently placed over water (with methylene blue to improve the visual analysis) and a picture was taken when their movement was stabilized (Figure 12(b–d). Both uncoated boats reached an equilibrium characterized by a partially submerged state without surpassing the water/air interface (Figure 12(b,c)), meaning a floating state, for which the bottom and the lateral parts of the boats were in contact with water. In contrast, the biomimetic boat (Figure 12d) perfectly floated over the water surface, and only the bottom surface was in contact with it (approximately 15% of the boat volume), where the keel of the boat is partially submerged with trapped air. This means that the biomimetic surface decreases the contact area of the boat with the fluid, therefore decreasing the drag force that the fluid can exhort over the structure [88], which implies a decrease in the thrust force exerted by the water proportional to the decrease in the submerged volume (~85%). This means that the biomimetic boat could carry on its surface a mass of water equivalent to 85% of its volume before being submerged in a position equivalent to the flat boat. This effect combined with the fact that air pockets exist inside the biomimetic microchannels (Figures 6e and 12d) reduces the resistance to motion of an object in the water.

### 3.5. Recyclability test

**Figure 13.**
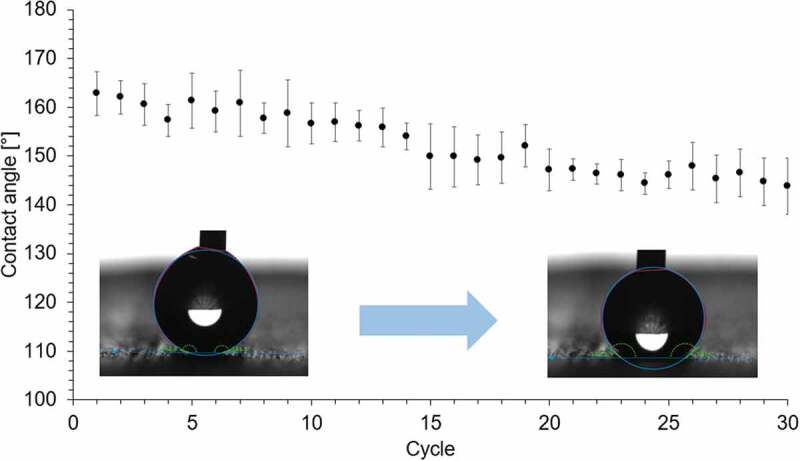
Recyclability experiment for the perpendicular contact angle of the biomimetic surface. a drop of water (4µl) was placed over the material 30 times (cycles).

The capability of the biomimetic material to maintain its wettability after being in contact with a drop of water was tested. A drop of water (4µL) was placed on the same spot over the biomimetic surface, then the perpendicular contact angle was measured, and the drop was removed being absorbed with paper. The process was repeated every 2minutes until 30 cycles were reached. This experiment was repeated on three different spots over the surface. The averaged results, presented in Figure 13, show that the surface goes from superhydrophobic to hydrophobic (contact angle >140°), as the surface decreases its contact angle 19°. As the printed structure remains the same, the change in the contact angle value is due to the detachment of the nanoparticles, which can be solved by a simple reapplication of the coating. Also, the wettability of the surface can be affected by the photocatalytic activity of the TiO2 nanoparticles (Figure 11c), so the material must be kept away from sunlight.

### 3.6. Drop bouncing dynamics

**Figure 14.**
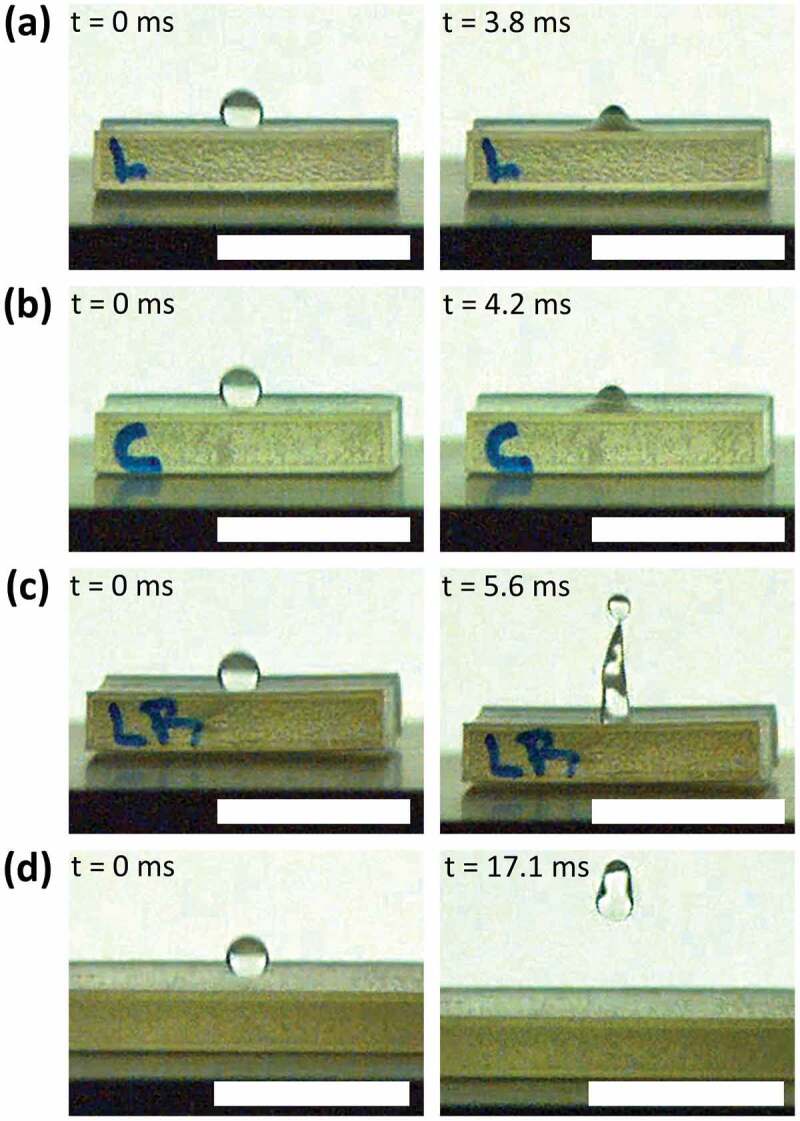
Drop bouncing dynamics comparison (first contact and highest position): (a) flat uncoated, (b) microchannels uncoated, (c) flat coated, and (d) microchannels coated (biomimetic). the drop, having a volume of 6µl (scale bar showing 10mm), falls from a height of 60mm (*We*=34), only bouncing off the biomimetic surface (d).

Drop bouncing dynamics defines the capability of a surface to repel water droplets during their impact as observed in practical applications such as self-cleaning, anti-icing, anti-corrosion, and water repellent surfaces in general [89]. A water drop, having a volume of 6µL, was released over the four different surfaces using the goniometer dispensation system, and then the bouncing dynamics were recorded at 2873 fps (see Supporting Video 4). The releasing height (*h*) of this experiment was varied from 20 to 100mm (distance from the tip of the needle to the surface), which translates to a range of Weber number ($We=\frac{\rho \nu^{2}D}{\sigma }$) between 11 and 57. Depending on the interactions between the water drop and the surface, two main processes can be observed: adhesion on the surface or a conventional bouncing. In the case of the flat-uncoated and microchannel-uncoated surfaces for all heights, and for the flat-coated surface for *h*>20, the drop adheres to the material. This behavior means that the material does not repel falling water drops. These results coincide with the observations made in the inclined surface experiments, where the drops do not roll over. Otherwise, in the case of the flat-coated surface with *h*=20, the drop bounces having a contact time of 14ms and a bounce dynamic, measured through the pancake quality, of *Q*=$\frac{D_{min}}{D_{max}}$=.5 (where *D*_*min*_ is the minimum jumping diameter and *D*_*max*_ is the maximum spreading diameter), which is in the range of conventional bouncing [90]). In the case of the microchannel-coated surface (biomimetic) for all the heights, the drop bounces off the material showing a conventional bouncing. The material therefore repels the fluid, where the drop spreads over the surface showing a pancake shape (*Q <.6*) during impact but regroups and jumps recovering the shape of a droplet. Figure 14 displays photograms showing the highest position reached by the drop during the bouncing dynamic in the case of *h*=60mm for the different surfaces, where the drop only bounces off the biomimetic surface (Figure 14d).

**Figure 15.**
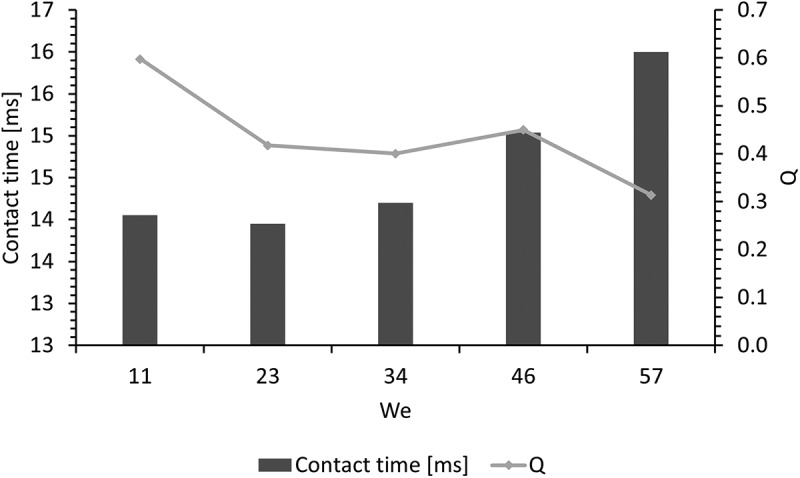
Contact time and pancake quality (*Q*) for the biomimetic surface (100µm microchannels coated), for drop releasing heights from 20 to 100mm (11 < *We* <57). Conventional bouncing is observed in all the experiments (*Q* <.6), where contact time increases with the impact velocity of the drop (6µl).

Figure 15 presents the contact time and pancake quality for each Weber number found during the test on the biomimetic material, which values coincide with previous studies [89–91]. When the velocity of the falling drop increases (higher heights), the contact time also increases, and the pancake quality decreases, due to the penetration of the fluid inside the air gaps that the biomimetic surface allows. An increase in the contact area implies an increment in the shear force over the drop’s surface, but the kinetic energy of the drop allows for a higher bounce. Because of this, the biomimetic surface repels falling drops at a range of Weber numbers. This observation, together with the previous dynamic experiments, shows that the new material has a promising prospect in dynamic water-repelling applications.

## 4. Conclusion

The combination of SLA 3D printed microchannels with a TiO_2_ -HTMS coating presented in this study can mimic the superhydrophobic behavior of the rice leaf. Our results show that apart from the designed channels and the presence of nanoparticles, the roughness generated by the SLA 3D printing filaments is relevant to adding a hierarchical structure to the material. On the flat printed surfaces, the presence of these filaments provides 35° of anisotropy and allows a hydrophobic wettability (contact angles measured >95°) in the perpendicular direction, and a hydrophilic behavior in the parallel direction (contact angles measured <74°). The TiO_2_-HTMS coating allowed to reach superhydrophobic states for all microchannel surfaces, showing contact angles >160°. The best biomimicry is obtained with 100µm coated microchannels with an advancing contact angle value of 165°, a contact angle hysteresis <9°, and a contact angle anisotropy of 5°, which meets the requirements of a rice leaf biomimetic superhydrophobic surface. This wettability was reached by a hierarchical three-level structure: printing filaments (around 10µm scale), microchannels (around 100µm scale), and nanometric coating (22–100nm scale). Air pockets under the drops placed over the biomimetic surface confirm the Cassie-Baxter state of the material. Dynamic tests show the self-cleaning property of the surface and its tunable wettability under UV-A irradiation. Floatability experiments show that the biomimetic surface can modify the contact area of an object placed over water. Numerical simulations confirm that the presence of air in a channel having a biomimetic bottom-wall increases the average velocity and decreases the friction factor, as a slip velocity exists on the biomimetic boundary layer. Our findings show that the combination of SLA 3D printing with a TiO_2_-HTMS coating is an excellent strategy to develop biomimetic superhydrophobic surfaces for future applications in fog-harvesting, anti-fouling, and drag reduction devices.

## Supplementary Material

Supplemental Material Video 4

Supplemental Material Video 3

Supplemental Material Video 2

Supplemental Material Video 1

## Data Availability

The data required to reproduce these findings can be found at: Barraza, Belen (2021), ‘M&D raw’, Mendeley Data, V2, doi: 10. 17,632/d33rk68dkj.2 and Barraza, Belen (2021), ‘M&D processed’, Mendeley Data, V2, doi: 10. 17,632/ptgf4w73cz.2

## References

[1] Chen C, Liu M, Zhang L, et al. Mimicking from rose petal to lotus leaf: biomimetic multiscale hierarchical particles with tunable water adhesion. ACS Appl Mater Interfaces. 2019;11(7):7431–7440. DOI:10.1021/acsami.8b21494 30699291

[2] Wu D, Wang JN, Wu SZ, et al. Three-Level biomimetic rice-leaf surfaces with controllable anisotropic sliding. Adv Funct Mater. 2011;21(15):2927–2932. DOI:10.1002/adfm.201002733

[3] Bixler GD, Bhushan B. Bioinspired rice leaf and butterfly wing surface structures combining shark skin and lotus effects. Soft Matter. 2012;8(44):11271–11284.

[4] Kumari N, Sood N, Krishnan V. Beetle wing inspired fabrication of nanojunction based biomimetic SERS substrates for sensitive detection of analytes. Mater Technol. 2020;1–12.

[5] Kumari N, Kumar A, Krishnan V. Ultrathin Au–Ag heterojunctions on nanoarchitectonics based biomimetic substrates for dip catalysis. J Inorg Organomet Polym Mater. 1902;31(5):1954–1966.

[6] Ariga K, Yamauchi Y. Nanoarchitectonics from atom to life. Chem – Asian J. 2020;15(6):718–728. 10.1002/asia.202000106 32017354

[7] Chen TL, Huang CY, Xie YT, et al. Bioinspired durable superhydrophobic surface from a hierarchically wrinkled nanoporous polymer. ACS Appl Mater Interfaces. 2019;11(43):40875–40885. DOI:10.1021/acsami.9b14325 31588736

[8] Bixler GD, Bhushan B. Fluid drag reduction and efficient self-cleaning with rice leaf and butterfly wing bioinspired surfaces. Nanoscale. 2013;5(17):7685–7710. 23884183 10.1039/c3nr01710a

[9] West N, Sammut K, Tang Y. Material selection and manufacturing of riblets for drag reduction: an updated review. Proce Inst Mech Eng Part L J Mat Des Appl. 2018;232:610–622.

[10] Bixler GD, Bhushan B. Bioinspired micro/nanostructured surfaces for oil drag reduction in closed channel flow. Soft Matter. 2013;9(5):1620–1635.

[11] Shen Y, Wu Z, Tao J, et al. Spraying preparation of eco-friendly superhydrophobic coatings with ultralow water adhesion for effective anticorrosion and antipollution. ACS Appl Mater Interfaces. 2020;12(22):25484–25493. DOI:10.1021/acsami.0c06074 32406672

[12] Wang H, He M, Liu H, et al. One-Step fabrication of robust superhydrophobic steel surfaces with mechanical durability, thermal stability, and anti-icing function. ACS Appl Mater Interfaces. 2019;11(28):25586–25594. DOI:10.1021/acsami.9b06865 31267735

[13] Souza JGS, Bertolini M, Costa RC, et al. Targeting pathogenic biofilms: newly developed superhydrophobic coating favors a host-compatible microbial profile on the titanium surface. ACS Appl Mater Interfaces. 2020;12(9):10118–10129. DOI:10.1021/acsami.9b22741 32049483

[14] Han K, Park TY, Yong K, et al. Combinational biomimicking of lotus leaf, mussel, and sandcastle worm for robust superhydrophobic surfaces with biomedical multifunctionality: antithrombotic, antibiofouling, and tissue closure capabilities. ACS Appl Mater Interfaces. 2019;11(10):9777–9785. DOI:10.1021/acsami.8b21122 30785265

[15] Jeevahan J, Chandrasekaran M, Britto Joseph G, et al. Superhydrophobic surfaces: a review on fundamentals, applications, and challenges. J Coatings Technol Res. 2018;15(2):231–250. DOI:10.1007/s11998-017-0011-x

[16] Tenjimbayashi M, Samitsu S, Watanabe Y, et al. Liquid marble patchwork on super-repellent surface. Adv Funct Mater. 2021;31:1–8.

[17] Jung YC, Bhushan B. Biomimetic structures for fluid drag reduction in laminar and turbulent flows. J Phys Condens Matter. 2010;22(3):035104. 21386280 10.1088/0953-8984/22/3/035104

[18] Lee SG, Lim HS, Lee DY, et al. Tunable anisotropic wettability of rice leaf-like wavy surfaces. Adv Funct Mater. 2013;23(5):547–553. DOI:10.1002/adfm.201201541

[19] Sharma V, Krishnan V. Fabrication of highly sensitive biomimetic SERS substrates for detection of herbicides in trace concentration. Sens Actuators B Chem. 2018;262:710–719.

[20] Fresnais J, Chapel JP, Poncin-Epaillard F. Synthesis of transparent superhydrophobic polyethylene surfaces. Surf Coat Technol. 2006;200(18–19):5296–5305.

[21] Wang S, Liu K, Yao X, et al. Bioinspired surfaces with superwettability: new insight on theory, design, and applications. Chem Rev. 2015;115(16):8230–8293. DOI:10.1021/cr400083y 26244444

[22] Dong Z, Vuckovac M, Cui W, et al. 3D printing of superhydrophobic objects with bulk nanostructure. Adv Mater. 2021;33(45):2106068. DOI:10.1002/adma.202106068 PMC11468021 34580937

[23] Lee KM, Park H, Kim J, et al. Fabrication of a superhydrophobic surface using a fused deposition modeling (FDM) 3D printer with poly lactic acid (PLA) filament and dip coating with silica nanoparticles. Appl Surf Sci. 2019;467-468:979–991.

[24] Milionis A, Noyes C, Loth E, et al. Superhydrophobic 3D printed surfaces by dip-coating. Tech Proc 2014 NSTI Nanotechnol Conf Expo, NSTI-Nanotech 2014. 2014;2:157–160.

[25] Milionis A, Noyes C, Loth E, et al. Water-repellent approaches for 3-D printed internal passages. Mater Manuf Process. 2016;31(9):1162–1170. DOI:10.1080/10426914.2015.1059443

[26] Zhang L, Wu J, Hedhili MN, et al. Inkjet printing for direct micropatterning of a superhydrophobic surface: toward biomimetic fog harvesting surfaces. J Mater Chem a. 2015;3(6):2844–2852. DOI:10.1039/C4TA05862C

[27] He Z, Chen Y, Yang J, et al. Fabrication of Polydimethylsiloxane films with special surface wettability by 3D printing. Compos Part B Eng. 2017;129:58–65.

[28] Yang Y, Li X, Zheng X, et al. 3D-Printed biomimetic super-hydrophobic structure for microdroplet manipulation and oil/water separation. Adv Mater. 2018;30:1–11. 10.1002/adma.201704912 29280219

[29] Jafari R, Cloutier C, Allahdini A, et al. Recent progress and challenges with 3D printing of patterned hydrophobic and superhydrophobic surfaces. Int J Adv Manuf Technol. 2019;103(1–4):1225–1238. DOI:10.1007/s00170-019-03630-4

[30] Zhang H, Li Y, Lu Z, et al. Differential expression of microRnas during fiber development between fuzzless-lintless mutant and its wild-type allotetraploid cotton. Sci Rep. 2017;7(1):3–10. DOI:10.1038/s41598-017-00038-6 28127052 PMC5428375

[31] Qin L, Chu Y, Zhou X, et al. Fast healable superhydrophobic material. ACS Appl Mater Interfaces. 2019;11(32):29388–29395. DOI:10.1021/acsami.9b07563 31313569

[32] Zhang X, Guo Y, Zhang Z, et al. Self-Cleaning superhydrophobic surface based on titanium dioxide nanowires combined with polydimethylsiloxane. Appl Surf Sci. 2013;284:319–323.

[33] Lu Y, Sathasivam S, Song J, et al. Robust self-cleaning surfaces that function when exposed to either air or oil. Science. 2015;347(6226):1132–1134. DOI:10.1126/science.aaa0946 25745169

[34] Heo KJ, Bin JS, Shin J, et al. Water-repellent TiO 2 -Organic dye-based air filters for efficient visible-light-activated photochemical inactivation against bioaerosols. Nano Lett. 2021;21(4):1576–1583. DOI:10.1021/acs.nanolett.0c03173 33275432

[35] Liu J, Ye L, Sun Y, et al. Elastic superhydrophobic and photocatalytic active films used as blood repellent dressing. Adv Mater. 2020;32(11):1908008. DOI:10.1002/adma.201908008 32009264

[36] Ding Y, Leng Y, Huang N, et al. Effects of microtopographic patterns on platelet adhesion and activation on titanium oxide surfaces. J Biomed Mater Res - Part a. 2013;101(3):A:622–632. DOI:10.1002/jbm.a.34361 22926985

[37] Nakajima A, Hashimoto K, Watanabe T, et al. Transparent superhydrophobic thin films with self-cleaning properties. Langmuir. 2000;16(17):7044–7047. DOI:10.1021/la000155k

[38] Yan C, Jiang P, Jia X, et al. 3D printing of bioinspired textured surfaces with superamphiphobicity. Nanoscale. 2020;12(5):2924–2938. DOI:10.1039/C9NR09620E 31993618

[39] Quan H, Zhang T, Xu H, et al. Photo-Curing 3D printing technique and its challenges. Bioact Mater. 2020;5(1):110–115. DOI:10.1016/j.bioactmat.2019.12.003 32021945 PMC6992881

[40] Li Y, Mao H, Hu P, et al. Bioinspired functional surfaces enabled by multiscale stereolithography. Adv Mater Technol. 2019;4:1–7.

[41] Peng L, Chen K, Chen D, et al. Study on the enhancing water collection efficiency of cactus-and beetle-like biomimetic structure using UV-induced controllable diffusion method and 3D printing technology. RSC Adv. 2021;11(24):14769–14776. DOI:10.1039/D1RA00652E 35424002 PMC8697806

[42] Bidkar RA, Leblanc L, Kulkarni AJ, et al. Skin-Friction drag reduction in the turbulent regime using random-textured hydrophobic surfaces. Phys Fluids. 2014;26(8):085108. DOI:10.1063/1.4892902

[43] Walsh MJ. Riblets as a viscous drag reduction technique. Aiaa J. 1983;21(4):485–486.

[44] García-Mayoral R, Jiménez J. Drag reduction by riblets. Philos Trans R Soc a Math Phys Eng Sci. 2011;369(1940):1412–1427. 10.1098/rsta.2010.0359 21382822

[45] Saravi SS, Cheng K. A review of drag reduction by riblets and micro-textures in the turbulent boundary layers. Eur Sci J. 2013;9:62–81.

[46] Moore AR, Lowson MV. Drag reduction in a rectangular duct using riblets. Aeronaut J. 1995;99:187–193.

[47] Abdulbari HA, Yunus RM, Abdurahman NH, et al. Going against the flow—a review of non-additive means of drag reduction. J Ind Eng Chem. 2013;19(1):27–36. DOI:10.1016/j.jiec.2012.07.023

[48] Daniello RJ, Waterhouse NE, Rothstein JP. Drag reduction in turbulent flows over superhydrophobic surfaces. Phys Fluids. 2009;21(8):1–9.

[49] FreeFEM - An open-source PDE solver using the finite element method.

[50] Bathe K-J. Finite element method. Wiley Encycl Comput Sci Eng. 2008;1–12.

[51] Reddy PD JN. Introduction to the finite element method. 4th ed. New York Chicago San FranciscoAthens London MadridMexico City Milan New DelhiSingapore Sydney Toronto: McGraw-Hill Education; 2019.

[52] Formlabs. Safety data sheet Clear Resin. 2019. p. 2.

[53] Förch R, Schönherr H, At J. Appendix C: Contact Angle Goniometry. In: Fçrch R Schçnherr H and ATAJ, editors. Surf Des Appl Biosci Nanotechnol. Weinheim: WILEY-VCH Verlag GmbH & Co.; 2009. p. 471–473.

[54] Song D, Daniello RJ, Rothstein JP. Drag reduction using superhydrophobic sanded Teflon surfaces. Exp Fluids. 2014;55(8):1783.

[55] Jung T, Choi H, Kim J. Effects of the air layer of an idealized superhydrophobic surface on the slip length and skin-friction drag. J Fluid Mech. 2016;790:R11–R112.

[56] Golovin KB, Gose J, Perlin M, et al. Bioinspired surfaces for turbulent drag reduction. Philos Trans R Soc a Math Phys Eng Sci. 2016;374:2073. 10.1098/rsta.2016.0189 PMC4928507 27354731

[57] Ou J, Rothstein JP. Direct velocity measurements of the flow past drag-reducing ultrahydrophobic surfaces. Phys Fluids. 2005;17(10):103606.

[58] Ou J, Perot B, Rothstein JP. Laminar drag reduction in microchannels using ultrahydrophobic surfaces. Phys Fluids. 2004;16(12):4635–4643.

[59] Cui J, Li W, Lam WH. Numerical investigation on drag reduction with superhydrophobic surfaces by lattice-boltzmann method. Comput Math with Appl. 2011;61(12):3678–3689.

[60] Zhang RL, Di QF, Wang XL, et al. Numerical study of the relationship between apparent slip length and contact angle by Lattice Boltzmann Method. J Hydrodyn. 2012;24(4):535–540. DOI:10.1016/S1001-6058(11)60275-8

[61] Choi CH, Ulmanella U, Kim J, et al. Effective slip and friction reduction in nanograted superhydrophobic microchannels. Phys Fluids. 2006;18(8):087105. DOI:10.1063/1.2337669

[62] El-Desoky MM, Morad I, Wasfy MH, et al. Synthesis, structural and electrical properties of PVA/TiO2 nanocomposite films with different TiO2 phases prepared by sol–gel technique. J Mater Sci Mater Electron. 2020;31(20):17574–17584. DOI:10.1007/s10854-020-04313-7

[63] Mishra V, Warshi MK, Sati A, et al. Investigation of temperature-dependent optical properties of TiO2 using diffuse reflectance spectroscopy. SN Appl Sci. 2019;1(3):1–8. DOI:10.1007/s42452-019-0253-6

[64] Khoee S, Bagheri Y, Hashemi A. Composition controlled synthesis of PCL–PEG Janus nanoparticles: magnetite nanoparticles prepared from one-pot photo-click reaction. Nanoscale. 2015;7(9):4134–4148. 25666985 10.1039/c4nr06590e

[65] León A, Reuquen P, Garín C, et al. FTIR and raman characterization of TiO2 nanoparticles coated with polyethylene glycol as carrier for 2-methoxyestradiol. Appl Sci. 2017;7(1):1–9. DOI:10.3390/app7010049

[66] Mugundan S, Rajamannan B, Viruthagiri G, et al. Synthesis and characterization of undoped and cobalt-doped TiO2 nanoparticles via sol–gel technique. Appl Nanosci. 2015;5(4):449–456. DOI:10.1007/s13204-014-0337-y

[67] Wanag A, Sienkiewicz A, Rokicka-Konieczna P, et al. Influence of modification of titanium dioxide by silane coupling agents on the photocatalytic activity and stability. J Environ Chem Eng. 2020;8(4):103917. DOI:10.1016/j.jece.2020.103917

[68] Xu L, Wang L, Shen Y, et al. Preparation of hexadecyltrimethoxysilane-modified silica nanocomposite hydrosol and superhydrophobic cotton coating. Fibers Polym. 2015;16(5):1082–1091. DOI:10.1007/s12221-015-1082-x

[69] Razmjou A, Mansouri J, Chen V. The effects of mechanical and chemical modification of TiO2 nanoparticles on the surface chemistry, structure and fouling performance of PES ultrafiltration membranes. J Memb Sci. 2011;378(1–2):73–84.

[70] Bachmann J, Gleis E, Fruhmann G, et al. Investigation of the temperature influence on the dual curing urethane-methacrylate resin Rigid Polyurethane 70 (RPU 70) in digital light synthesis (DLS). Addit Manuf. 2021;37.

[71] What does resolution mean in 3D printing? [Internet]. [cited 2021 May 31]. Available from: https://formlabs.com/blog/horizontal-resolution-meaning-3d-printing/.

[72] Wang X, Cai X, Guo Q, et al. I3DP, a robust 3D printing approach enabling genetic post-printing surface modification. Chem Commun. 2013;49(86):10064–10066. DOI:10.1039/c3cc45817b 24002351

[73] Tricinci O, Terencio T, Mazzolai B, et al. 3D Micropatterned surface inspired by Salvinia molesta via Direct Laser Lithography. ACS Appl Mater Interfaces. 2015;7(46):25560–25567. DOI:10.1021/acsami.5b07722 26558410 PMC4667276

[74] Barahman M, Lyons AM. Ratchetlike slip angle anisotropy on printed superhydrophobic surfaces. Langmuir. 2011;27(16):9902–9909. 21699191 10.1021/la201222a

[75] Fürstner R, Barthlott W, Neinhuis C, et al. Wetting and self-cleaning properties of artificial superhydrophobic surfaces. Langmuir. 2005;21(3):956–961. DOI:10.1021/la0401011 15667174

[76] Riedel J, Vucko MJ, Blomberg SP, et al. Skin hydrophobicity as an adaptation for self-cleaning in geckos. Ecol Evol. 2020;10(11):4640–4651. DOI:10.1002/ece3.6218 32551049 PMC7297746

[77] Dalawai SP, Saad Aly MA, Latthe SS, et al. Recent advances in durability of superhydrophobic self-cleaning technology: a critical review. Prog Org Coatings. 2020;138:105381.

[78] Sharma V, Balaji R, Krishnan V. Fog-harvesting properties of dryopteris marginata: role of interscalar microchannels in water-channeling. 10.3390/biomimetics3020007 PMC6352672 31105229

[79] Sharma V, Orejon D, Takata Y, et al. Gladiolus dalenii based bioinspired structured surface via soft lithography and its application in water vapor condensation and fog harvesting. ACS Sustain Chem Eng. 2018;6(5):6981–6993. DOI:10.1021/acssuschemeng.8b00815

[80] Wu Y, Shen Y, Tao J, et al. Facile spraying fabrication of highly flexible and mechanically robust superhydrophobic F-SiO 2 @PDMS coatings for self-cleaning and drag-reduction applications. New J Chem. 2018;42(22):18208–18216. DOI:10.1039/C8NJ04275F

[81] Luan K, He M, Xu B, et al. Spontaneous directional self-cleaning on the feathers of the aquatic bird anser cygnoides domesticus induced by a transient superhydrophilicity. Adv Funct Mater. 2021;2010634:1–9.

[82] Panchanathan D, Kwon G, Qahtan TF, et al. Kinetics of photoinduced wettability switching on nanoporous titania surfaces under oil. Adv Mater Interfaces. 2017;4(21):1–10. DOI:10.1002/admi.201700462

[83] Feng R, Song F, Xu C, et al. A quadruple-biomimetic surface for spontaneous and efficient fog harvesting. Chem Eng J. 2021;422:130119.

[84] Shi R, Tian Y, Wang L. Bioinspired fibers with controlled wettability: from spinning to application. ACS Nano. 2021;15(5):7907–7930. 33909405 10.1021/acsnano.0c08898

[85] Bhushan B. Biomimetics. 3rd ed. Columbus, OH: Springer US; 2018.

[86] McHale G, Shirtcliffe NJ, Evans CR, et al. Terminal velocity and drag reduction measurements on superhydrophobic spheres. Appl Phys Lett. 2009;94(6):1–6. DOI:10.1063/1.3081420

[87] Dong H, Cheng M, Zhang Y, et al. Extraordinary drag-reducing effect of a superhydrophobic coating on a macroscopic model ship at high speed. J Mater Chem a. 2013;1(19):5886–5891. DOI:10.1039/c3ta10225d

[88] Munson B, Young D, Okiishi T, et al. Fundamentals of fluid mechanics. Sixth ed. Welter J Dumas S, editors. USA: Wiley; 2009.

[89] Liu Y, Moevius L, Xu X, et al. Pancake bouncing on superhydrophobic surfaces. Nat Phys 2014 107 [Internet]. 2014 [[cited 2022 Mar 26]];10:515–519. Available from: https://www.nature.com/articles/nphys2980 10.1038/nphys2980 PMC5444522 28553363

[90] Guo C, Zhao D, Sun Y, et al. Droplet impact on anisotropic superhydrophobic surfaces. Langmuir. 2018;34(11):3533–3540. DOI:10.1021/acs.langmuir.7b03752 29436832

[91] Baggio M, Weigand B. Numerical simulation of a drop impact on a superhydrophobic surface with a wire. Phys Fluids [Internet]. 2019;31(11):112107. doi:10.1063/1.5123593.

